# Prediction of clinically relevant adverse drug events in surgical patients

**DOI:** 10.1371/journal.pone.0201645

**Published:** 2018-08-23

**Authors:** Jacqueline M. Bos, Gerard A. Kalkman, Hans Groenewoud, Patricia M. L. A. van den Bemt, Peter A. G. M. De Smet, J. Elsbeth Nagtegaal, Andre Wieringa, Gert Jan van der Wilt, Cornelis Kramers

**Affiliations:** 1 Department of clinical pharmacy, Canisius Wilhelmina hospital, Nijmegen, the Netherlands; 2 Department of health evidence, Radboud university medical center, Nijmegen, the Netherlands; 3 Department of hospital pharmacy, Erasmus university medical center, Rotterdam, the Netherlands; 4 Department of pharmacy, Radboud university medical center, Nijmegen, the Netherlands; 5 Department Scientific Institute for Quality of Healthcare, Radboud university medical center, Nijmegen, the Netherlands; 6 Department of clinical pharmacy, Meander Medical Center, Amersfoort, the Netherlands; 7 Department of clinical pharmacy, Isala hospital, Zwolle, the Netherlands; 8 Department of clinical pharmacology and toxicology, Radboud university medical center, Nijmegen, the Netherlands; Universita degli Studi di Padova, ITALY

## Abstract

**Background:**

Risk stratification of hospital patients for adverse drug events would enable targeting patients who may benefit from interventions aimed at reducing drug-related morbidity. It would support clinicians and hospital pharmacists in selecting patients to deliver a more efficient health care service. This study aimed to develop a prediction model that helps to identify patients on the day of hospital admission who are at increased risk of developing a clinically relevant, preventable adverse drug event during their stay on a surgical ward.

**Methods:**

Data of the pre-intervention measurement period of the P-REVIEW study were used. This study was designed to assess the impact of a multifaceted educational intervention on clinically relevant, preventable adverse drug events in surgical patients. Thirty-nine variables were evaluated in a univariate and multivariate logistic regression analysis, respectively. Model performance was expressed in the Area Under the Receiver Operating Characteristics. Bootstrapping was used for model validation.

**Results:**

6780 admissions of patients at surgical wards were included during the pre-intervention period of the PREVIEW trial. 102 patients experienced a clinically relevant, adverse drug event during their hospital stay. The prediction model comprised five variables: age, number of biochemical tests ordered, heparin/LMWH in therapeutic dose, use of opioids, and use of cardiovascular drugs. The AUROC was 0.86 (95% CI 0.83–0.88). The model had a sensitivity of 80.4% and a specificity of 73.4%. The positive and negative predictive values were 4.5% and 99.6%, respectively. Bootstrapping generated parameters in the same boundaries.

**Conclusions:**

The combined use of a limited set of easily ascertainable patient characteristics can help physicians and pharmacists to identify, at the time of admission, surgical patients who are at increased risk of developing ADEs during their hospital stay. This may serve as a basis for taking extra precautions to ensure medication safety in those patients.

## Introduction

Pharmacotherapy is one of the most commonly applied interventions in hospital healthcare. In addition to the beneficial effects, prescribing medication also introduces risks of medication errors and adverse drug events that can lead to potentially preventable morbidity, mortality, and costs [1–3]. Patients on surgical wards are particularly at risk, due to their need for pain medication and antibiotics, frequent adjustments of antithrombotic regimens, and blood and fluid loss. In the case of elderly surgical patients, multiple co-morbidities requiring multiple drugs contribute even more to potential drug-related problems [4].

Risk prediction is a routine component in daily care practice in both specific areas (e.g. approaches used to determine stroke risk in patients with atrial fibrillation) as well as more generally, for example in identifying patients likely to require hospital admission. Risk stratification of hospital patients for adverse drug events (ADE) can target a population that can benefit from interventions aimed to reduce drug-related morbidity, as a form of personalized medicine. It can support clinicians and hospital pharmacists in patient prioritization to deliver more efficient health care service [5]. In a systematic review Yourman et al. emphasized that failure to consider risk prediction in a clinical setting can result in poor patient outcomes [6].

A recent review identified four studies that developed and validated ADE risk-prediction tools for use in adults over 65 years of age [4, 5, 7–9]. These prediction models had poor to modest performance and did not address clinical impact, thereby limiting clinical usefulness. Because a large number of variables contribute to ADE occurrence in patients, it is impossible to precisely predict every ADE in every patient. Therefore, Stevenson et al. suggested that these risk prediction strategies should focus either on one specific harmful ADE (e.g. gastrointestinal or intracranial bleeding) or ADEs in patients with a particular illness or clinical characteristic, for instance surgical patients [5]. In addition, since the aim is to prevent patient harm, it seems more rational to predict clinically relevant, potentially preventable adverse events, instead of adverse reactions in general.

The P-REVIEW study (Pharmacist-led Risk patients medication EValuation to Initiate Event reduction on surgical Wards) was designed to determine whether a multifaceted intervention comprised of educating the prescriber combined with medication review and pharmaceutical visits to the ward by the hospital pharmacist could lead to a reduction of adverse drug events among surgical patients [10]. In this study, experts assessed clinically relevant, potentially preventable ADE in a cohort of 13,264 admissions of surgical patients. An ADE was classified as clinically relevant when it was fatal, life-threatening, disabling, incapacitating, or when it resulted in or prolonged hospitalisation. When an ADE resulted from suboptimal care, it was classified as potentially preventable. The study showed a significant benefit for patients in the intervention period. To improve the cost effectiveness of medication review and other measures to prevent avoidable harm, it would be useful to identify patients at risk of clinically relevant ADEs in the surgical ward. For this purpose, we used the P-REVIEW data to develop a risk-prediction model that could identify patients at risk of a clinically relevant, potentially preventable adverse drug event during admission to the surgical ward, on the day of hospital admission.

## Materials and methods

### Study setting and population

The P-REVIEW study is a prospective open intervention study designed to investigate whether a multifaceted educational intervention could lead to a reduction of clinically relevant, potentially preventable adverse drug events among patients in surgical wards. The study was performed in two large general teaching hospitals in the Netherlands and has been described in detail elsewhere [10]. Patients who were admitted to the surgical, urological, or orthopaedic ward of one of the two hospitals during a period of six months were included. In case of readmission, patients could be included more than once. Day care patients were excluded. For the development of the prediction model, data were used from patients during the pre-intervention period.

### P-REVIEW data set

Data available in the P-REVIEW dataset were collected for each admission, including patient characteristics, drug history, and biochemical, haematological, and microbiological markers. The major part of the data collection was performed automatically from the EHR (Electronic Health Record). In addition, some of the data were collected manually by a review of medical records.

Data from the day of admission of each patient was extracted. If more laboratory values were available of the same variable, the last one, being the most recent value available, was extracted.

Assessment of clinically relevant, potentially preventable adverse drug events that led to death, temporary or permanent disability, increased length of hospital stay, or readmission within 30 days was performed in the P-REVIEW study by teams of experts consisting of a hospital pharmacist and a hospital-based physician, who were not affiliated with the study hospitals. To classify seriousness the National Coordinating Council for Medication Error Reporting and Prevention (NCC MERP) index was used. Categories E to I of this index were considered to be clinically relevant. The causality between prescription error and the drug related problem was assessed using the algorithm by Kramer et al. The potential preventability was assessed using the algorithm according to Schumock et al., modified by Lau et al. [10].

### Predicting variables

From the data that were collected in the P-REVIEW study, candidate model parameters were selected on the basis of reports in the literature of their association with ADEs [1–4, 11–18]. Thirty-nine risk factors were identified, including patient characteristics (age, gender), department of admission (general surgery versus orthopaedic surgery and urology), type of admission (emergency versus elective); medication (number of medications, use of gastrointestinal drugs, hypoglycemic drugs, vitamin K antagonists, heparin or low molecular weight heparin (LMWH) in therapeutic dose, thrombocyte aggregation inhibitors, cardiovascular drugs in general, cardiac drugs, diuretics, betablockers, renin angiotensin system (RAS) inhibitors, antilipaemica, corticosteroids, antimicrobials, chemotherapy, non-steroidal anti-inflammatory drugs (NSAIDs), opioids, antiepileptics, central nervous system (CNS) agents in general, antipsychotics, anxiolytics, antidepressants, and serious drug-drug interactions); laboratory test results (albumin, glucose, hemoglobin, international normalized ratio (INR), potassium, sodium, leucocytes, chronic kidney disease epidemiology (CKD-EPI), oxygen saturation, positive microbiological blood culture, number of biochemical tests ordered (<20 versus ≥20)) [1–4, 11–18]. Drugs were coded according to the Anatomical Therapeutic and Chemical codes [19]. The glomerular filtration rate was computed by the Chronic Kidney Disease Epidemiology Collaboration formula (CKD-EPI)[20]. Missing data where imputed using the ‘multiple imputation’ procedure from SPSS version 22. SPSS uses a Markov Chain Monte Carlo (MCMC) algorithm known as Fully Conditional Specification (FCS). This method can be used when the pattern of missing data is arbitrary. For each iteration and for each variable in the order specified in the variable list, the FCS method fits a univariate (single dependent variable) model using all other available variables in the model as predictors. Linear regression was used to predict a scale variable and logistic regression to predict categorical variables. Variables of which more than 60% of data were missing were left out of the analysis.

### Model development

Model development consisted of two stages [21]. In the first stage, possible predictors were tested using a univariate binary logistic regression model. Variables that were found to be statistically significant (P<0.05) were taken forward to the next stage of multivariate analysis.

In case of variables that in clinical practice can be either too high or too low and confer risk in both situations, categorization of variables was performed (for instance, low potassium values < 3.5 mmol/l, normal values between 3.5 and 5.0 mmol/l and high values > 5.0 mmol/l).

In the second stage, backward and forward elimination procedures were used in multivariate logistic regression analysis in order to detect the best predictors. The removal criterion was set at p = 0.10.

Results from the univariate and multivariate logistic regression models were expressed in terms of the odds ratio with 95% confidence intervals and p-values.

Standardized odd ratios were computed to allow for comparison of the strength of the association between the various continuous variables and the probability of an ADE. Standardization was achieved through Z-transformation.

### Model performance

Model performance of the logistic regression model was expressed in the Area Under the Curve (AUC) as computed by a Receiver Operating Characteristics curve analysis (ROC analysis) using the probability as predicted by the regression model and the real outcome (ADE).

### Model validation

Bootstrapping was used to assess the internal validity of the model. Two hundred bootstrap samples were drawn to assess the reliability of the model expressed in over-optimism and the uniform shrinkage factor.

Statistical analyses were performed using SPSS Statistics version 22 (IBM Software, New York). Bootstrapping was performed in SAS version 9.4.

### Ethics

For all stages of this research, patient records were anonymized prior to analysis in accordance with prevailing privacy regulations.

The institutional review boards of the Isala Hospital and the Meander Medical Center in the Netherlands stated that the study was exempt from ethical approval.

## Results

The pre-intervention period of the P-REVIEW dataset study population comprised 6780 admissions of 5940 patients at surgical wards. A clinically relevant, potentially preventable adverse drug event during hospital stay, which led to death, temporary or permanent disability, increased length of hospital stay or readmission within 30 days was determined in 102 patients. The most frequent types of events were haemorrhage (19), arterial or venous thrombosis(7), renal insufficiency, dehydration or electrolyte related events (13), drug intoxication in renal insufficiency (4), central nervous system events (48) and faecal impaction (11). Characteristics of patients who did, and those who did not experience an ADE during hospital stay are shown in Table 1.

**Table 1:** Patient characteristics.

	Admissions with a clinically relevant ADE(n = 102)	Admissions without a clinically relevant ADE(n = 6678)
Mean age of patients in years ± SD	78.7 ± 8.7	63.1 ± 17.6
Gender of patients, n (%) female	50(49.0%)	3331 (49.9%)
Department of admission, n (%)		
General surgery	67 (65.7%)	3824 (57.3%)
Urology	4 (3.9%)	1244 (18.6%)
Orthopedic surgery	31 (30.4%)	1610 (24.1%)
Admission, n (%) elective	39 (38.2%)	4194 (62.8%)
No. of medications (mean ± SD)	11.1 ± 4.8	6.6 ± 5.5

### Univariate analysis

The candidate predictive variables of a clinically relevant ADE during hospital stay and results of the univariate analysis are reported in Table 2.

**Table 2:** Candidate predictive variables.

		Univariate analysis			Standardized OR
Predictive variables(references)	N missing (%)	OR	CI	P-value	
***Patient characteristics***					
Age (years)	0 (0)	1.09	1.07–1.09	<0.0001	4.33 (3.11–6.03)
Gender (m/f)	0 (0)	0.97	0.65–1.43	0.862	
Department of admission	204 (3.1)			0.004	
General Surgery vs Urology		4.95	1.79–13.65	0.002	
Orthopedic S vs Urology		5.89	2.07–16.28	0.001	
Admission (emergency vs elective)	235 (3.5)	2.91	1.94–4.37	<0.0001	
***Medication use (ATC-code)***					
No. of medications	0 (0)	1.13	1.10–1.16	<0.0001	1.94 (1.65–2.29)
Serious drug-drug interactions^b^	0 (0)	3.99	2.20–7.24	<0.0001	
Gastrointestinal drugs (A02)	0 (0)	1.60	1.08–2.37	0.019	
Hypoglycemics (A10)	0 (0)	3.17	2.04–4.93	<0.0001	
Vitamin K antagonists (B01AA)	0 (0)	2.03	1.04–3.90	0.038	
Heparin/LMWH in therapeutic dose (B01AB)	0 (0)	4.23	2.37–7.55	<0.0001	
Thrombocyte aggregation inhibitors (B01AC)	0 (0)	3.21	2.14–4.82	<0.0001	
Cardiovascular drugs (C)	0 (0)	9.31	5.29–16.38	<0.0001	
Cardiac drugs (C01)	0 (0)	3.55	2.23–5.65	<0.0001	
Diuretics (C03)	0 (0)	3.70	2.50–5.49	<0.0001	
Betablockers (C07)	0 (0)	3.35	2.26–4.76	<0.0001	
RAS inhibitors (C09)	0 (0)	3.15	2.12–4.66	<0.0001	
Antilipaemicae (C10)	0 (0)	2.36	1.58–3.55	<0.0001	
Corticosteroids (H02)	0 (0)	2.01	0.92–4.38	0.078	
Antimicrobials (J01,J02)	0 (0)	1.73	1.17–2.56	0.006	
Chemotherapy (L01)	0 (0)	NA			
NSAIDs (M01A)	0 (0)	0.71	0.45–1.13	0.149	
Opioids (N02A)	0 (0)	4.39	2.90–6.65	<0.0001	
Antiepileptics (N03)	0 (0)	1.59	0.69–3.67	0.273	
CNS agents (N05/N06)	0 (0)	4.91	2.70–8.94	<0.0001	
Antipsychotics (N05A)	0 (0)	1.65	1.10–2.48	0.015	
Anxiolytics (N05B)	0 (0)	1.59	0.84–2.99	0.153	
Antidepressants (N06A)	0 (0)	1.71	0.93–3.14	0.086	
***Laboratory data***					
Albumin (g/L)	5839 (86.1)	0.96	0.92–1.00	0.055	0.74(0.54–1.01)
Glucose (mmol/L)	5097 (75.2)	1.14	1.04–1.24	0.004	1.40(1.11–1.77)
Hemoglobin (mmol/L)(low vs normal and high)^a^	3901 (57.5)	2.83	1.48–5.43	0.002	
INR (ratio)	5775 (85.2)	1.07	0.84–1.36	0.582	1.08(0.83–1.39)
Potassium (mmol/L)	3085 (45.5)			0.640	
(low vs. normal)		0.95	0.43–2.07	0.888	
(high vs. normal)^a^		1.74	0.54–5.65	0.355	
Sodium (mmol/L)	3184 (47.0)			0.046	
(low vs. normal)		2.06	1.17–3.65	0.013	
(high vs. normal)^a^		1.11	0.15–8.21	0.916	
Leucocytes (10^9^/L)	3784 (55.8)	0.98	0.92–1.04	0.475	0.91(0.69–1.19)
CKD-EPI (ml/min/1.73 m^2^)	3062 (45.1)			0.001	
(severely impaired vs. normal)		2.70	1.26–5.80	0.010	
(moderately impaired vs. normal))^a^		2.25	1.40–3.64	0.001	
Oxygen saturation (%)	6322 (93.2)	NA			
Positive microbiological blood culture	0 (0)	NA			
Number of biochemical tests (≥20 versus <20)	0 (0)	3.63	2.45–5.37	<0.0001	1.36(1.24–1.48)

Abbreviations: OR, Odds Ratio; CI, confidence interval; GS, general surgery; OS, orthopedic surgery; U, urology; LMWH, low molecular weight heparin; CNS, central nervous system; INR, international normalized ratio; NA, not applicable/computable;

a. Values for normal ranges were defined as follows: hemoglobin normal values: male 8,5–11,0 mmol/l, female 7,5–10,0 mmol/l; potassium normal values: 3.5–5.0 mmol/l; sodium normal values: 135–145 mmol/l; CKD-EPI: normal values >60, moderately impaired renal failure 30–60, severely impaired renal failure <30 ml/min/1.73 m^2^.

b. Serious drug-drug interactions were defined as having the potential to cause long-lived residual symptoms or handicap, failure of life-saving therapy or death. These interactions were identified using the Dutch national database known as ‘G-Standard’.

### Multivariate analysis

The significant variables (p<0.05) were identified from the univariate analysis and taken forward to the next stage.

In all 20 imputed datasets, both backward and forward elimination procedure in multivariate analysis identified the same 5 variables that were significantly associated with the risk of developing a clinically relevant adverse drug event. It appeared that these 5 predictor variables did not have missing values. Therefore, the final analysis could be performed with the original, complete dataset.

The coefficients (ß), standard errors (SE), and odds ratios (OR) with 95% confidence intervals of the variables are shown in Table 3. The final model comprised age, number of biochemical tests ordered, heparin/LMWH in therapeutic dose, use of opioids, and use of cardiovascular drugs at the time of hospital admission.

**Table 3:** Coefficients, standard errors and Odds Ratios (OR) with 95% confidence intervals (CI) of the five variables of the final model. **The OR as found after applying the shrinkage factor is also given**.

	Logistic regression			
	ß	SE	OR (95% CI)	OR after applying the shrinkage factor
Age	0.060	.10	1.062 (1.039–1.083)	1.059
Number of biochemical tests. (≥20 tests versus <20 tests)	0.770	.208	2.159 (1.181–2.841)	2.094
Heparin/LMWH in therapeutic dose (Y/N)	0.604	.307	1.830 (1.786–3.819)	1.786
Cardiovascular drugs (Y/N)	1.261	.220	3.529 (1.786–5.902)	3.355
Opioids (Y/N)	0.785	.300	2.192 (1.272–3.278)	2.125

Abbreviations: ß, coefficient; CI, confidence interval; LMWH, low molecular weight heparin; OR,Odds Ratio; SE, standard error;

**Fig 1:**
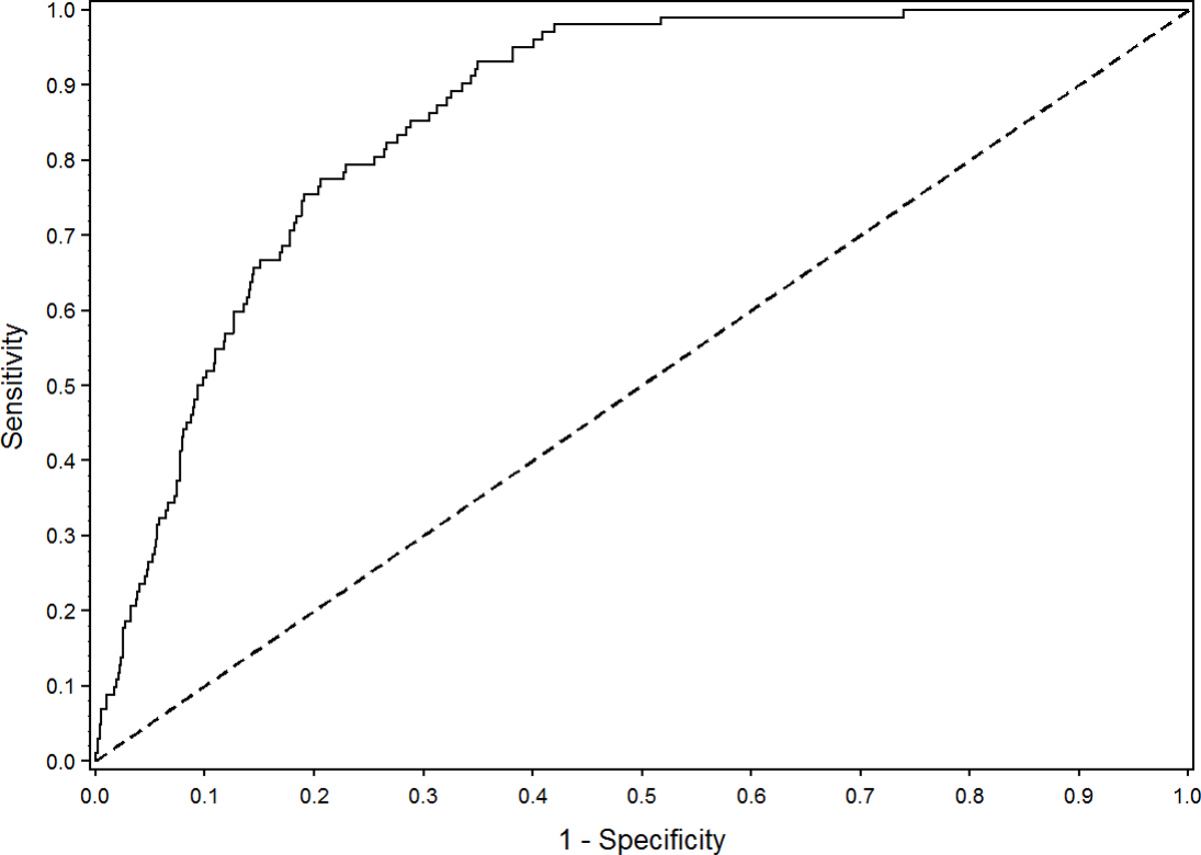
The ROC curve of the model.

The AUC of the model was 0.858 (95% CI 0.831–0.884). The ROC curve is shown in Fig 1. The validation of the model using bootstrap samples showed an over-optimism of 0.008 and a shrinkage factor of 0.960, leading to a corrected performance of the model of 0.85 (Table 3).

When the cut-off point for a high risk of developing an ADE was set at 1.6%, the model showed a sensitivity of 80.4% and a specificity of 73.4%. At this cut-off level, positive and negative predictive value were 4.5% and 99.6%, respectively.

The formula of the logistic regression model that allows for calculating the individual risk of a clinically relevant adverse drug event to a surgical patient is shown below.

Formula individual risk = 1/(1 + exp(-1*LP))

Where LP is the so called Linear Predictor which for our model is defined as:

Linear Predictor = 10.082 +

0.060 * age (in years) +

0.770 (in case number of biochemical tests ordered ≥ 20) +

0.604 * heparin/LMWH (in therapeutic dose) +

1.261 (in case the patient uses cardiovascular drugs) +

0.785 (in case the patient uses opioids)

## Discussion

The risk prediction model resulting from this study helps to identify surgical patients that are at increased risk of sustaining a clinically relevant potentially preventable adverse drug event. This model uses five clinical variables that can be obtained routinely on hospital admission, and that can be incorporated into clinical practice as a tool to target patients that can benefit from interventions aimed at reducing potentially preventable clinically relevant adverse drug events during hospital stay in surgical wards.

The model was developed using data from the pre-intervention phase of the P-REVIEW study, which assessed clinically relevant, potentially preventable ADE among patients at surgical wards [10]. This is the first study that developed a risk prediction model focusing on clinically relevant potentially preventable ADE instead of adverse drug reactions in general. This study only targeted surgical patients, which leads to a better model performance. We only included variables that are available on the first day after hospital admission to be able to operationalize a model to predict risk during hospital stay, immediately after hospital admission. Therefore, variables such as length of hospital stay, interdisciplinary consultation or admission to the intensive care unit during hospital stay, were not included in our analysis.

Our final model contains five variables; age of the patient, number of biochemical tests ordered, treatment with heparin/LMWH in therapeutic dose, treatment with opioids, and treatment with cardiovascular drugs. Our model shows an acceptable level of fit and discrimination performance. We can use the model not only to label patients at risk of experiencing a drug-related event but also to label patients that are very unlikely to experience an event. The negative predictive value of our model is very high. Therefore, one could use this model to also identify patients for whom automated computerized clinical decision support without surveillance by the hospital pharmacist is sufficient.

An overview of studies of development and validation of risk prediction models for ADR or ADE is shown in Table 4. Other studies showed different predictive variables and found an area under the receiver operator characteristic curve (AUROC), varying between 0.70–0.74, with relatively low sensitivity and specificity scores. We hypothesized that focusing on patient cohorts with restricted clinical characteristics (surgical patients) and focusing on clinically relevant, potentially preventable adverse drug events, instead of adverse drug reactions in general, would lead to a better performing prediction model.

**Table 4:** Overview studies of development and validation of risk prediction models for ADR or ADE.

Study (year)	Setting and country	Sample size and population	Outcome	Predicting variables in the model	Model performance	Model validation
McElnay et al. (1997)(7)	General hospital, United Kingdom	929 patients(> 65 years old)	ADE	Digoxine Antidepressiants Gastrointestinal disorders Chronic obstructive airways disease Angina Abnormal potassium level Thinks drugs are responsible for hospital admission	AUROC: not presented	204 patients Sensitivity 41% Specificity 69%
Trivalle et al. (2011)(9)	Geriatric rehabilitation centers, France	526 patients	ADE	Number of medications Antipsychotic treatment Recent anticoagulant use	AUROC: 0.70	Bootstrapping
Tangiisuran et al. (2014)(8)	Teaching hospital, United Kingdom	690 patients (>85 years old)	ADR	Hyperlipidemia Number of medications > 8 Length of stay > 12 days Antidiabetic drugs Elevated white cell count	AUROC: 0.74 Sensitivity 80% Specificity 55%	483 patients AUROC:0.73 Sensitivity 84% Specificity 43%
Onder et al. (2010)(4)	Community and university-based hospitals, Italy	5936 elderly patients	ADR	Number of medications History of ADR Heart failure Liver disease Presence of 4 or more co-morbidities Renal failure	AUROC: 0.71 Sensitivity: 68% Specificity: 65%	483 patients AUROC: 0.70
The present study	Teaching hospitals, the Netherlands	6780 admissions of 5940 surgical patients	Clinically relevant, potentially preventable ADE	Age Number of biochemical tests ordered Heparin/LMWH in therapeutic dose Cardiovascular drugs Opioids	AUROC: 0.86 Sensitivity: 80% Specificity: 73%	Bootstrapping AUROC: 0.85

Abbreviations: ADE: adverse drug event; ADR: adverse drug reaction; AUROC: Area under the Receiver Operator Characteristic Curve; LMWH: low molecular weight heparin.

In our study, the strongest independent risk factor was the age of the patients. As shown in many other studies, advancing age is associated with an increased number of comorbidities in association with polypharmacy resulting in an increased risk of ADEs [2, 3, 13].

This is the first study that explores the number of biochemical tests ordered as a predictive factor for drug related events. We hypothesized that besides the use of specific laboratory values as electronic triggers, the number of biochemical tests might also be useful in identifying a risk of ADE during hospital admission. Before diagnosing the patient’s condition, the physician may suspect a possible adverse outcome. This suspicion alerts the physician and leads to increased laboratory orders to clarify the patient’s condition and to prevent serious problems. Moreover, deviant laboratory values will lead to further monitoring [22]. Consequently, the number of biochemical tests might be a useful electronic trigger to identify a patient at risk of ADE, pointing out an acute unstable clinical situation [16, 23]. In our study this factor has proven to be a strong predictor for drug related problems in surgical wards.

Surgery in patients who use anticoagulation therapy, is a challenge. Guidelines on perioperative management of anticoagulation are complicated and people are at risk of either bleeding or thrombosis as a result of the surgical procedures. For patients with a high risk of thromboembolism, measured with a CHA_2_DS_2_-VASC score, needing certain surgical procedures, it is necessary to perform bridging with LMWH at therapeutic dose during the perioperative period. If this procedure is not correctly performed, these patients are at high risk of serious ADEs. Pardo Carbello et al. already demonstrated that the most frequent fatal ADE is haemorrhage [24].

We found that use of cardiovascular drugs and opioids are significant risk factors in surgical patients. Cardiovascular drugs are generally used by a vulnerable older population with multiple co-morbidities. Use of opioids often indicates (temporary) severe morbidity. The literature has shown that patients using these drugs have an increased risk of experiencing ADEs [15, 25].

This study has several strengths. The P-REVIEW database contains information on clinically relevant potentially preventable drug related events (leading to death, temporary or permanent disability, increased hospital stay or readmission) in a very large cohort. Aiming at prevention of patient harm, it seems more rational to focus on clinically relevant, potentially preventable adverse events, instead of adverse reactions in general. We focused on patients in surgical wards. By studying patient cohorts with restricted clinical characteristics the performance of risk prediction models will improve. Furthermore, we used variables that can be routinely obtained on hospital admission, so implementation can easily be operationalized.

There are some limitations to our study. In our analysis, we used potentially predictive variables that were available in the P-REVIEW database. Mostly, these data were automatically obtained from hospital databases [10]. The lack of automated coded information for some variables limited the use of these variables, e.g. information on co-morbidities using the International Classification of Diseases (ICD-10), frailty or cognitive impairment, reason for hospital admission or type of surgery performed. We internally validated our model by bootstrapping but we have not yet been able to externally validate this model in clinical practice. Because patients could be included more than once, in case of readmission, this may have led to an immortal time bias. However, since only a small patients were admitted several times in the study, we think this bias was very limited.

The development of our risk prediction model has potentially important implications for clinical practice and research. By using this model, lower-risk patients could be managed less extensively (for instance, only automatically using CPOE/CDSS), whereas higher risk patients could receive more intensive interventions, such as medication review, aimed at reducing drug-related adverse outcomes. Such selective use of ancillary precautions could also help to improve the cost-effectiveness of medication safety interventions. In that way, this risk model, which combines clinical and medication related variables can guide clinical intervention, delivered as part of an integrated system built on the principles of medication safety [5]. Future research should confirm whether intensive pharmacovigilance of high risk patients really leads to less adverse drug events in hospitalized patients. Our risk model can be incorporated into a CPOE system and thereby generate automatic risk-evaluation based on patients’ medical records upon hospital admission. Above a pre-specified cut-off point, the score can assist hospital pharmacists or prescribing physicians in their decisions to review the patient’s medication or to perform other relevant interventions. Under the cut-off point (not necessarily the same cut-off point), it may be possible for the hospital pharmacist to prioritize efforts in medication safety interventions and rely, when possible, on an automatic medication safety system.

## Conclusions

A risk prediction model was developed to identify surgical patients at risk of experiencing a clinically relevant, potentially preventable adverse drug event during hospital admission. The resulting model contains five variables: age of the patient, number of biochemical tests ordered, treatment with heparin/LMWH in therapeutic dose, treatment with opioids, and treatment with cardiovascular drugs. This model can be used to guide the hospital pharmacist and the physician to effectively and efficiently implement clinical interventions to improve medication safety.
